# Role of Transvaginal Ultrasound and MRI in the Evaluation and Characterization of Gynaecological Pelvic Pathologies: A Clinico-Histopathological Correlation Study

**DOI:** 10.7759/cureus.117037

**Published:** 2026-09-27

**Authors:** Ashish A Bhalodiya, Pradeep A Maheshwari, Dharaben I Zalavadiya, Kalpesh Zanzrukiya, Vijay N Vaidya

**Affiliations:** 1 Department of Radiodiagnosis, Gujarat Medical Education and Research Society (GMERS) Medical College, Vadodara, IND; 2 Department of Radiology, Sardar Patel Medical College and Research Centre, Ahmedabad, IND; 3 Department of Community Medicine, Gujarat Medical Education and Research Society (GMERS) Medical College, Vadodara, IND; 4 Department of Forensic Medicine, Smt. B. K. Shah Medical Institute and Research Centre, Vadodara, IND; 5 Department of Radiology, Gujarat Medical Education and Research Society (GMERS) Medical College, Vadodara, IND

**Keywords:** corelation, diagnostic accuracy, magnetic resonance imaging (mri), pelvic pathology, ultrasonography (usg)

## Abstract

Introduction

Transvaginal USG is the first-line imaging modality for evaluating female pelvic pathologies because of its accessibility and cost-effectiveness. However, indeterminate findings and operator dependence may limit its diagnostic accuracy. MRI, with its superior soft-tissue contrast and multiplanar capability, serves as an important complementary modality for lesion characterization and staging.

Aim

To compare the diagnostic performance of USG and MRI in the evaluation of female pelvic pathologies using clinical, surgical, and histopathological findings as the reference standard.

Materials and methods

This prospective study included 50 women (11-70 years) with clinically suspected pelvic pathology who were evaluated between July 2013 and April 2014. All patients underwent transabdominal and transvaginal USG with Doppler, followed by pelvic MRI on a 3.0-T scanner using standard T1-, T2-, diffusion-weighted, and contrast-enhanced sequences where indicated. Imaging findings were compared with histopathological findings, intraoperative findings, or clinical follow-up. Diagnostic accuracy parameters and Cohen's kappa were calculated.

Results

Fibroids were the most common pathology (26%), followed by cervical carcinoma (16%) and endometrial carcinoma (10%). MRI reduced the proportion of indeterminate lesions from 12% on USG to 2% and accurately identified the organ of origin in nearly all cases. MRI demonstrated 100% sensitivity and specificity for fibroids, adenomyosis, dermoid cysts, simple cysts, and endometriotic cysts, whereas USG showed lower sensitivity for fibroids (84.6%), dermoid cysts (50%), and the three malignant pathologies evaluated (cervical, endometrial, and ovarian carcinoma; USG sensitivity, 75-80%; MRI sensitivity, 100%). Agreement between modalities ranged from good to perfect (κ = 0.648-1.000), with very good agreement for fibroids (κ = 0.891) and malignant lesions (κ = 0.834-0.878).

Conclusion

MRI showed numerically higher diagnostic accuracy and improved lesion characterization compared with USG, particularly for complex pelvic lesions and equivocal ultrasound findings, although these differences were not evaluated using a formal paired statistical comparison. While USG remains the preferred first-line investigation, MRI is an invaluable complementary modality that improves diagnostic confidence and facilitates optimal clinical management.

## Introduction

Imaging is essential in evaluating gynaecological pelvic pathology, and transvaginal ultrasound (TVUS) and MRI each play a distinct and complementary role in this process. TVUS is the first-line imaging tool for the female pelvis because it is widely available, low-cost, and allows real-time assessment. High-resolution TVUS provides good diagnostic accuracy for detecting and characterizing most adnexal and uterine lesions. However, it has some limitations, including a limited field of view, interference from bowel gas, and strong dependence on the operator's skill and experience. These limitations mean that a significant number of findings remain indeterminate on ultrasound alone [1].

MRI is increasingly used as a second-line, problem-solving tool because of its high tissue contrast, ability to image in multiple planes, and lack of ionizing radiation. When an adnexal mass cannot be clearly classified on ultrasound, which occurs in roughly 18-31% of cases, MRI-based scoring systems such as the Ovarian-Adnexal Reporting and Data System (O-RADS) MRI score have shown high sensitivity and specificity for distinguishing benign from malignant lesions. This helps guide decisions regarding surgery, monitoring, or fertility-preserving treatment [1].

A similar pattern is seen with other common gynaecological conditions. For uterine fibroids and adenomyosis, MRI is more sensitive and specific than TVUS and is particularly useful for identifying coexisting conditions and planning surgery [2]. For deep infiltrating endometriosis, both modalities perform similarly in detecting rectosigmoid and uterosacral ligament disease, although MRI is better for evaluating disease spread across multiple compartments [3]. For suspected Müllerian duct anomalies, MRI is considered a reference standard because it clearly demonstrates uterine zonal anatomy, which is important because surgical management differs depending on the exact type of anomaly [4].

Overall, current evidence supports a stepwise approach: ultrasound as the initial imaging test, followed by MRI for further characterization, staging, or resolution of uncertain findings. However, studies that directly correlate imaging results with clinical and histopathological findings in the same group of patients, across a broad range of pelvic pathologies rather than a single condition, are still limited. This study was designed to help address that gap.

## Materials and methods

This prospective study was conducted over 10 months (July 2013-April 2014) in the Department of Imaging and Radiodiagnosis, Grant Medical Foundation, Ruby Hall Clinic, Pune. Grant Medical Foundation is the charitable trust that owns and operates Ruby Hall Clinic, while Poona Medical Research Foundation is a closely affiliated body that has historically served as the Institutional Ethics Committee for research and clinical trials conducted at this hospital; both names refer to the same single study site. Fifty female patients (aged 11-70 years) with clinically suspected pelvic pathology were included. The study protocol was approved by the Institutional Ethics Committee, Poona Medical Research Foundation (approval number: PS-35-040). Written informed consent was obtained from all adult participants prior to enrolment; for participants younger than 18 years, written parental/legal guardian consent and participant assent were obtained, as applicable. Patients were recruited using a consecutive sampling method, wherein all women presenting to the Department of Imaging and Radiodiagnosis with clinically suspected pelvic pathology during the study period and fulfilling the inclusion criteria were enrolled; no formal a priori sample-size calculation was performed, as this was designed as a descriptive diagnostic-accuracy study across a broad spectrum of pelvic pathologies. Patients with amenorrhea and positive pregnancy tests, clinical suspicion of ectopic pregnancy, or contraindications to MRI were excluded.

All patients underwent transabdominal and transvaginal ultrasonography (Philips IU-22 (Philips Ultrasound, Bothell, Washington, USA)) using curvilinear (C5-1), linear (L9-3), and endovaginal (C9-5) probes, with colour Doppler assessment of lesion vascularity. MRI was subsequently performed in all cases as a complementary problem-solving modality following ultrasound, and the reporting radiologist had access to the prior USG findings, consistent with routine clinical workflow; formal blinded, independent interpretation of the two modalities was not performed, and this is acknowledged as a limitation of the study. MRI was performed on a 3.0 T Philips Ingenia scanner (Philips Medical Systems Nederland B.V., Best, The Netherlands) using a phased-array coil, with the field of view extending from the aortic bifurcation to the pubic symphysis. Patients fasted for 4 hours prior to imaging and received IV hyoscine butylbromide to reduce bowel peristalsis. Sequences included axial/sagittal/coronal FSE T1- and T2-weighted, T2 3D, DWI (b800), and pre-/post-contrast mDIXON and dynamic eTHRIVE sequences (gadopentetate dimeglumine, 0.1 mmol/kg), performed where indicated. Lesions were characterized by organ of origin, morphology, tissue content, vascularity, and demarcation, using histopathology, intraoperative findings, or clinical/imaging follow-up as the reference standard.

Data were entered into Microsoft Excel (Microsoft Corporation, Redmond, WA, USA) and analysed using MedCalc Statistical Software (MedCalc Software Ltd, Ostend, Belgium); the sensitivity and specificity of USG and MRI were calculated for pathologies with ≥2 cases using 2×2 contingency tables, and intermodality agreement was assessed using the kappa statistic. Each patient contributed a single dominant pelvic lesion for analysis, with no patient having more than one concurrent pathology recorded; diagnostic accuracy analysis was therefore performed on a per-patient basis, which was numerically equivalent to a per-lesion analysis in this cohort.

## Results

A total of 50 women with clinically suspected pelvic pathologies were enrolled in the study. The majority of patients were between 41 and 50 years of age (32%), followed by those aged 31-40 years (24%), 61-70 years (18%), 21-30 years (12%), 51-60 years (10%), and 11-20 years (4%) (Table 1). The spectrum of final diagnoses demonstrated that fibroids were the most common pathology, accounting for 13 (26%) cases, followed by carcinoma of the cervix in 8 (16%), carcinoma of the endometrium in 5 (10%), and both dermoid cyst and carcinoma of the ovary in 4 (8%) patients each. Hemorrhagic cysts were identified in 3 (6%) patients, while adenomyosis, simple cyst, and endometriotic cyst each accounted for 2 (4%) cases. Endometrial hyperplasia, polycystic ovary syndrome (PCOS), tubo-ovarian mass, carcinoma of the vagina, uterine agenesis, septate uterus, and bicornuate uterus were observed in one patient (2%) each.

**Table 1:** Baseline characteristics and spectrum of pelvic pathologies. PCOS: Polycystic ovarian syndrome.

Variable	n (%)
Age distribution (years)	
11-20	2 (4)
21-30	6 (12)
31-40	12 (24)
41-50	16 (32)
51-60	5 (10)
61-70	9 (18)
Final diagnosis (n = 50)	
Fibroid	13 (26)
Carcinoma of the cervix	8 (16)
Carcinoma of the endometrium	5 (10)
Dermoid cyst	4 (8)
Carcinoma of the ovary	4 (8)
Hemorrhagic cyst	3 (6)
Adenomyosis	2 (4)
Simple cyst	2 (4)
Endometriotic cyst	2 (4)
Endometrial hyperplasia	1 (2)
PCOS	1 (2)
Tubo-ovarian mass	1 (2)
Carcinoma of the vagina	1 (2)
Uterine agenesis	1 (2)
Septate uterus	1 (2)
Bicornuate uterus	1 (2)
Characterization of lesions	
Final benign	32 (64)
Final malignant	18 (36)
Organ of origin	
Uterus	24 (48)
Ovary	16 (32)
Cervix	8 (16)
Vagina	1 (2)
Tubo-ovarian	1 (2)

The reference standard was applied hierarchically according to each patient's clinical management: histopathological examination of surgical or biopsy specimens was used in 31 (62%) cases, comprising all malignant lesions and resected benign lesions; intraoperative findings at surgery or laparoscopy were used in 6 (12%) cases, including subserosal fibroids confirmed at myomectomy/hysterectomy and Müllerian anomalies confirmed at laparoscopy; clinical and imaging follow-up, based on resolution or stability of the lesion on serial ultrasound, was used in 11 (22%) cases, mainly hemorrhagic cysts, simple cysts, fibroids, and adenomyosis managed conservatively; and clinical, hormonal, and laboratory correlation was used in 2 (4%) cases (congenital absence of the uterus and polycystic ovarian syndrome).

Among the 13 uterine fibroids, intramural fibroids constituted the majority (61%), followed by subserosal fibroids (31%) and submucosal fibroids (8%). Among the 17 true adnexal-organ lesions (ovarian and tubo-ovarian in origin), dermoid cyst (Figure 1) and carcinoma of the ovary (Figure 2) were the most frequent diagnoses (23.5% each), followed by hemorrhagic cyst (17.6%), simple cyst and endometriotic cyst (11.8% each), and PCOS and tubo-ovarian mass (5.9% each). In addition, four subserosal fibroids, though uterine in origin, were exophytic and closely mimicked adnexal masses on imaging, representing a distinct diagnostic challenge in organ-of-origin characterization discussed separately below. Overall, 18 patients (36%) had malignant lesions, of which carcinoma of the cervix represented the largest proportion (44%) (Figure 3), followed by carcinoma of the endometrium (28%), carcinoma of the ovary (22%), and carcinoma of the vagina (6%). Staging of cervical carcinoma revealed that International Federation of Gynecology and Obstetrics stage IIB (FIGO stage IIB) was the most common stage at presentation (37.5%), followed by stage IB (25%), while stages IIA, IIIA, and IVA each accounted for 12.5% of cases.

**Figure 1:**
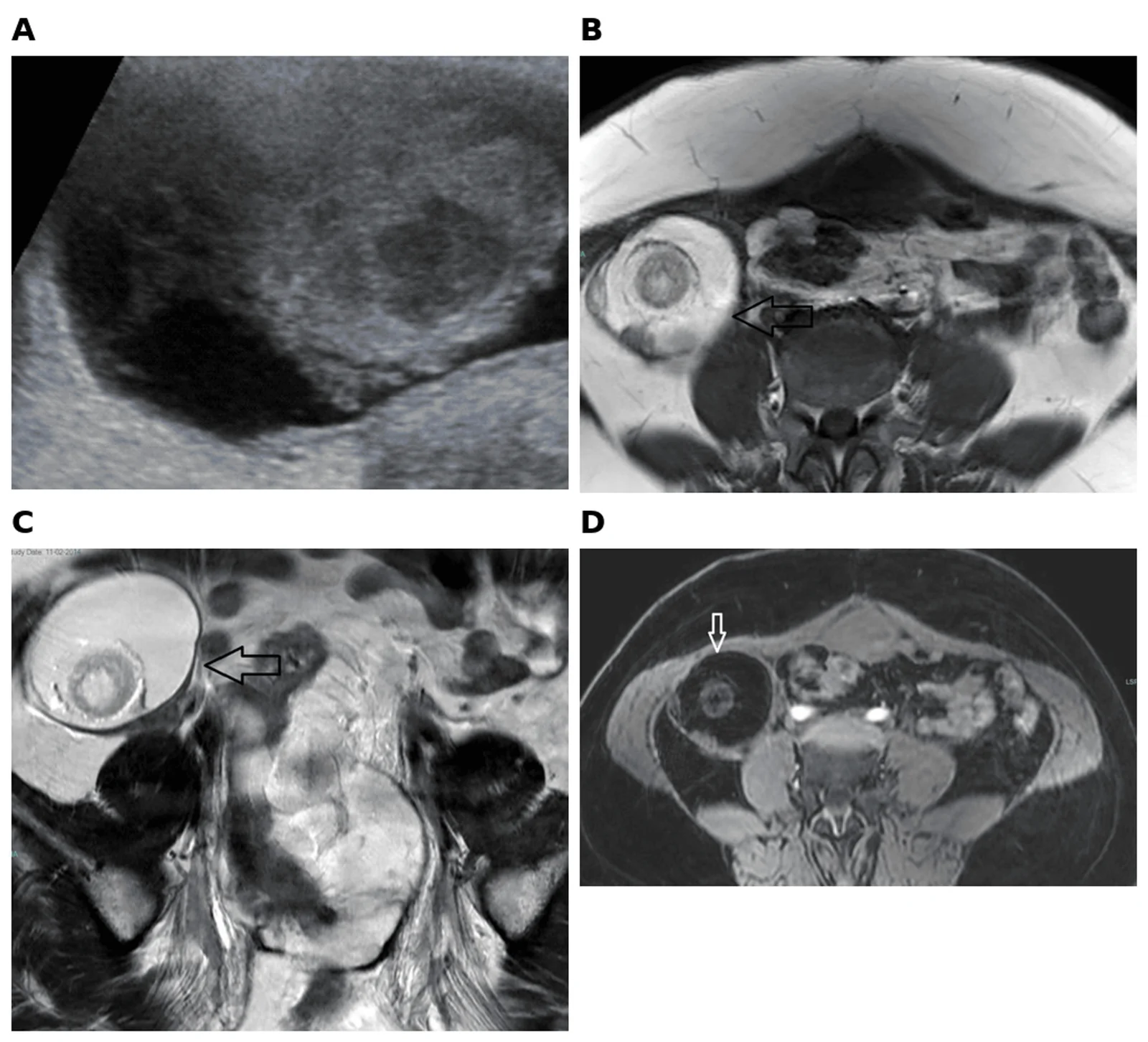
Dermoid cyst. (A) TAS showed a solid-cystic adnexal mass. The solid component showed a hypoechoic nodule within. (B) Axial T1WI showed a hyperintense lesion, similar to fat signal intensity, with an isointense solid component in the centre. (C) Coronal T2WI showed a hyperintense lesion, similar to fat signal intensity, with a hypointense nodule within. (D) Fat-only axial mDIXON image demonstrated the fat component of the lesion. These findings were consistent with a dermoid cyst. TAS: Transabdominal ultrasonography; T1WI: T1-weighted imaging; T2WI: T2-weighted imaging; mDIXON: Modified Dixon.

**Figure 2:**
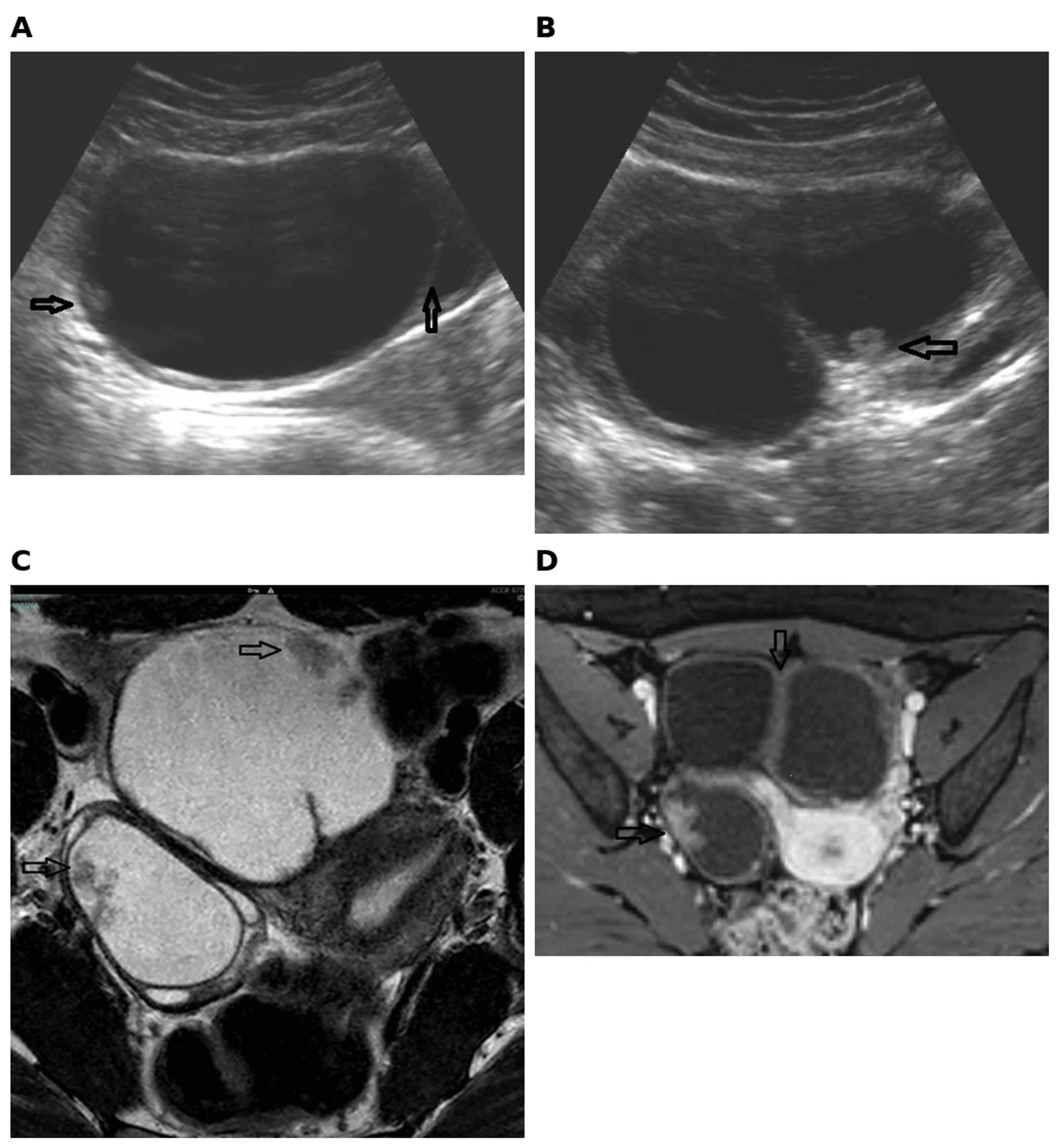
Ovarian carcinoma. (A) and (B) TAS of an adolescent girl showed bilateral large complex cystic lesions with small hyperechoic nodules and thick septa within. (C) Axial T2WI showed a large hyperintense cystic lesion with small hypointense nodules and multiple septations within the lesion. (D) Post-contrast fat-saturated axial mDIXON images showed enhancing solid mural nodules and enhancing septa. TAS: Transabdominal ultrasonography; T2WI: T2-weighted imaging; mDIXON: Modified Dixon.

**Figure 3:**
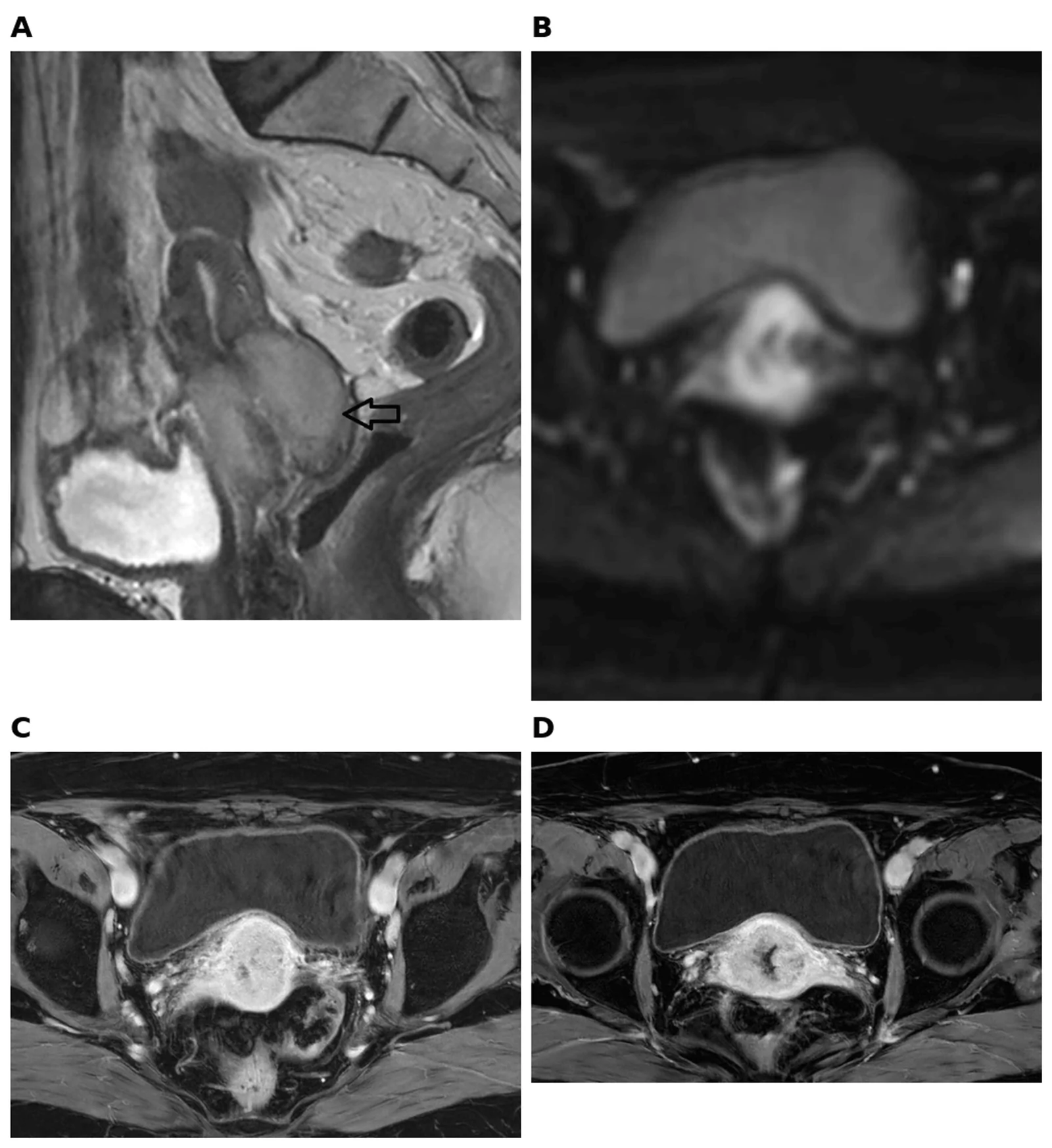
Cervical carcinoma, FIGO stage IIB. (A) Sagittal T2WI showed a hyperintense mass lesion involving the uterine cervix and extending to the lower uterine segment. (B) DWI (b = 800 s/mm²) showed a hyperintense mass with low ADC values. (C) and (D) Post-contrast axial mDIXON images showed enhancement of the mass with extension into the bilateral parametria. There was no involvement of the lateral pelvic wall or urinary bladder wall. FIGO: International Federation of Gynecology and Obstetrics; T2WI: T2-weighted imaging; DWI: Diffusion-weighted imaging; ADC: Apparent diffusion coefficient; mDIXON: Modified Dixon.

Regarding lesion characterization, USG classified 30 (60%) lesions as benign, 6 (12%) as indeterminate, and 14 (28%) as malignant. In comparison, MRI characterized 31 (62%) lesions as benign, 1 (2%) as indeterminate, and 18 (36%) as malignant, closely matching the final diagnosis. MRI substantially reduced the proportion of indeterminate lesions from 12% with USG to 2%, indicating improved lesion characterization; however, this difference was interpreted descriptively.

MRI also demonstrated excellent performance in determining the organ of origin. Based on the final diagnosis, lesions originated from the uterus in 24 (48%) patients, ovary in 16 (32%), cervix in 8 (16%), vagina in 1 (2%), and tubo-ovarian region in 1 (2%) patient. USG incorrectly classified three adnexal lesions, whereas MRI showed better agreement with the final organ-of-origin classification.

The diagnostic performance of USG and MRI for individual pelvic pathologies is summarized in Table 2. For uterine fibroids, USG demonstrated a sensitivity of 84.62%, specificity of 100%, positive predictive value (PPV) of 100%, and negative predictive value (NPV) of 94.87%, whereas MRI achieved 100% sensitivity, specificity, PPV, and NPV. For adenomyosis, MRI demonstrated 100% sensitivity, specificity, PPV, and NPV; USG showed 100% sensitivity but 97.92% specificity and 66.67% PPV, reflecting one case in which USG suggested adenomyosis that was not confirmed by MRI or the final diagnosis. In the diagnosis of dermoid cysts, MRI identified all cases with 100% sensitivity, while USG detected only half of the lesions (sensitivity, 50%), although specificity remained 100% for both modalities. Conversely, for hemorrhagic cysts, USG demonstrated 100% sensitivity, whereas MRI showed a lower sensitivity of 66.67%; however, specificity and PPV remained 100% for both techniques, with MRI maintaining a high NPV (97.92%). Both USG and MRI accurately diagnosed all cases of simple cysts and endometriotic cysts, yielding 100% sensitivity, specificity, PPV, and NPV for both pathologies.

**Table 2:** Diagnostic performance of USG and MRI compared with the final diagnosis. PPV: Positive predictive value; NPV: Negative predictive value; κ: Cohen’s kappa coefficient.

Pathology	Imaging modality	Sensitivity (%)	Specificity (%)	PPV (%)	NPV (%)	Cohen’s κ (95% CI)
Fibroid	USG	84.62	100	100	94.87	0.891 (0.743-1.000)
	MRI	100	100	100	100	
Adenomyosis	USG	100	97.92	66.67	100	0.790 (0.391-1.000)
	MRI	100	100	100	100	
Dermoid cyst	USG	50	100	100	95.83	0.648 (0.200-1.000)
	MRI	100	100	100	100	
Hemorrhagic cyst	USG	100	100	100	100	0.790 (0.391-1.000)
	MRI	66.67	100	100	97.92	
Simple cyst	USG	100	100	100	100	1.000 (1.000-1.000)
	MRI	100	100	100	100	
Endometriotic cyst	USG	100	100	100	100	1.000 (1.000-1.000)
	MRI	100	100	100	100	
Cervical carcinoma	USG	75	100	100	95.45	0.834 (0.610-1.000)
	MRI	100	100	100	100	
Endometrial carcinoma	USG	80	100	100	97.83	0.878 (0.641-1.000)
	MRI	100	100	100	100	
Ovarian carcinoma	USG	75	100	100	97.87	0.847 (0.550-1.000)
	MRI	100	100	100	100	

Agreement between USG and MRI was assessed using Cohen's kappa (κ) statistic. Perfect agreement was observed for simple cysts and endometriotic cysts (κ = 1.000). Agreement was very good for fibroids (κ = 0.891; 95% CI: 0.743-1.000) and good for adenomyosis (κ = 0.790; 95% CI: 0.391-1.000), hemorrhagic cysts (κ = 0.790; 95% CI: 0.391-1.000), and dermoid cysts (κ = 0.648; 95% CI: 0.200-1.000). Very good agreement was also observed for carcinoma of the endometrium (κ = 0.878; 95% CI: 0.641-1.000) and carcinoma of the ovary (κ = 0.847; 95% CI: 0.550-1.000), and good agreement was observed for carcinoma of the cervix (κ = 0.834; 95% CI: 0.610-1.000). Collectively, these findings indicate that MRI showed numerically higher point-estimate sensitivity and fewer indeterminate diagnoses than USG for several complex pelvic lesions; however, as this study was not designed or powered for formal paired statistical comparison between the two modalities, these differences should be interpreted descriptively rather than as evidence of statistically confirmed superiority.

## Discussion

The present prospective study compared the diagnostic performance of USG and MRI in the evaluation of female pelvic pathologies. The findings demonstrate that MRI showed numerically higher diagnostic performance, improved lesion characterization, more accurate determination of the organ of origin, and fewer indeterminate diagnoses than USG. These observations reinforce the complementary role of MRI as a problem-solving imaging modality, particularly when ultrasonographic findings are equivocal or when complex pelvic masses are encountered. As no formal paired statistical comparison was performed, these differences should be interpreted descriptively.

The demographic profile of our study population revealed that the majority of women were between 41 and 50 years of age, which is consistent with the age group in which benign uterine disorders, particularly leiomyomas, as well as gynaecological malignancies are frequently encountered. Fibroids represented the most common pathology (26%), followed by cervical and endometrial carcinomas. This distribution is consistent with global epidemiological data, in which uterine leiomyomas remain the most common benign pelvic tumours in women of reproductive and perimenopausal age [5], while cervical and endometrial cancers remain among the most frequently diagnosed gynaecological malignancies worldwide [6].

An important finding of the present study was the reduction in indeterminate lesions from 12% with USG to only 2% with MRI. This highlights the higher soft-tissue contrast of MRI, allowing more accurate differentiation of benign and malignant lesions. MRI correctly classified 36% of lesions as malignant, corresponding exactly with the final diagnosis, whereas USG underestimated malignant disease in several patients. Similar observations have been reported by Hricak H et al., who demonstrated that MRI provides superior soft-tissue characterization and staging capability compared with ultrasound because of its multiplanar imaging and excellent contrast resolution [7]. The European Society of Urogenital Radiology also recommends MRI as the preferred second-line imaging technique whenever ultrasound findings are inconclusive [8].

Accurate identification of the organ of origin is essential for treatment planning, especially in women presenting with large pelvic masses. In the present study, MRI correctly identified the origin of nearly all lesions, whereas USG misclassified three adnexal masses. Distinguishing ovarian masses from pedunculated subserosal fibroids or tubo-ovarian lesions can be particularly challenging on ultrasound because of its limited field of view and operator dependence. MRI overcomes these limitations through multiplanar imaging and superior delineation of anatomical planes, leading to improved surgical planning and avoidance of unnecessary procedures [9, 10].

Among benign lesions, uterine fibroids were the predominant pathology, with intramural fibroids accounting for more than 60% of cases. MRI achieved 100% sensitivity and specificity for fibroid detection, whereas USG demonstrated slightly lower sensitivity (84.6%). Although ultrasound remains the first-line investigation owing to its accessibility and cost-effectiveness, MRI provides greater accuracy in identifying the number, size, location, and degeneration of fibroids, particularly in women being considered for fertility-preserving surgery or uterine artery embolization. Similarly, MRI demonstrated perfect diagnostic performance for adenomyosis, consistent with a previous prospective study showing MRI to be an accurate non-invasive modality for diagnosing adenomyosis based on junctional zone assessment [11].

MRI demonstrated higher sensitivity than USG for diagnosing dermoid cysts, correctly identifying all cases, whereas ultrasound detected half of the lesions. Mature cystic teratomas may occasionally exhibit atypical sonographic appearances, while MRI reliably identifies intralesional fat and characteristic signal suppression on fat-saturated sequences, resulting in improved diagnostic confidence [12]. Interestingly, ultrasound demonstrated slightly higher sensitivity than MRI for hemorrhagic cysts in the present study. This finding is likely attributable to the small sample size rather than true superiority of ultrasound, as previous studies have shown both modalities to be highly accurate, with MRI primarily reserved for lesions that remain indeterminate after sonography [13].

The present study also demonstrated good to perfect agreement between USG and MRI for the benign lesions evaluated. Perfect agreement was observed for simple cysts and endometriotic cysts (κ = 1.000), while very good agreement was observed for fibroids (κ = 0.891). Good agreement was observed for adenomyosis (κ = 0.790), hemorrhagic cysts (κ = 0.790), and dermoid cysts (κ = 0.648). These findings suggest that ultrasound performs well for straightforward benign conditions, whereas MRI provides incremental value in complex, atypical, or diagnostically uncertain cases. Similar conclusions have been reported in the American College of Radiology (ACR) Appropriateness Criteria, which recommend MRI for characterization of indeterminate adnexal masses and comprehensive evaluation of pelvic malignancies [14].

The strengths of this study include prospective data collection, comparison with final clinical and histopathological diagnoses, and assessment of multiple pelvic pathologies within a single cohort. However, several limitations should be acknowledged. The relatively small sample size, particularly for uncommon lesions, limits the precision of sensitivity estimates for individual pathologies. Furthermore, this was a single-centre study, which may restrict the generalizability of the findings. Larger multicentre studies with standardized MRI protocols are warranted to validate these results and evaluate the cost-effectiveness of incorporating MRI into routine diagnostic algorithms.

## Conclusions

In conclusion, MRI showed numerically higher diagnostic performance than ultrasonography for several pelvic pathologies in this cohort, with improved lesion characterization, better assessment of the organ of origin, and fewer indeterminate diagnoses. While ultrasonography remains the preferred first-line imaging modality because of its availability and affordability, MRI serves as an important complementary investigation for complex pelvic lesions, suspected malignancies, and equivocal ultrasound findings.
